# Low-Carbohydrate Diets and All-Cause Mortality: A Systematic Review and Meta-Analysis of Observational Studies

**DOI:** 10.1371/journal.pone.0055030

**Published:** 2013-01-25

**Authors:** Hiroshi Noto, Atsushi Goto, Tetsuro Tsujimoto, Mitsuhiko Noda

**Affiliations:** 1 Department of Diabetes and Metabolic Medicine, Center Hospital, National Center for Global Health and Medicine, Tokyo, Japan; 2 Department of Diabetes Research, Diabetes Research Center, Research Institute, National Center for Global Health and Medicine, Tokyo, Japan; University of Chieti, Italy

## Abstract

**Objective:**

Low-carbohydrate diets and their combination with high-protein diets have been gaining widespread popularity to control weight. In addition to weight loss, they may have favorable short-term effects on the risk factors of cardiovascular disease (CVD). Our objective was to elucidate their long-term effects on mortality and CVD incidence.

**Data sources:**

MEDLINE, EMBASE, ISI Web of Science, Cochrane Library, and ClinicalTrials.gov for relevant articles published as of September 2012. Cohort studies of at least one year’s follow-up period were included.

**Review methods:**

Identified articles were systematically reviewed and those with pertinent data were selected for meta-analysis. Pooled risk ratios (RRs) with 95% confidence intervals (CIs) for all-cause mortality, CVD mortality and CVD incidence were calculated using the random-effects model with inverse-variance weighting.

**Results:**

We included 17 studies for a systematic review, followed by a meta-analysis using pertinent data. Of the 272,216 people in 4 cohort studies using the low-carbohydrate score, 15,981 (5.9%) cases of death from all-cause were reported. The risk of all-cause mortality among those with high low-carbohydrate score was significantly elevated: the pooled RR (95% CI) was 1.31 (1.07–1.59). A total of 3,214 (1.3%) cases of CVD death among 249,272 subjects in 3 cohort studies and 5,081 (2.3%) incident CVD cases among 220,691 people in different 4 cohort studies were reported. The risks of CVD mortality and incidence were not statistically increased: the pooled RRs (95% CIs) were 1.10 (0.98–1.24) and 0.98 (0.78–1.24), respectively. Analyses using low-carbohydrate/high-protein score yielded similar results.

**Conclusion:**

Low-carbohydrate diets were associated with a significantly higher risk of all-cause mortality and they were not significantly associated with a risk of CVD mortality and incidence. However, this analysis is based on limited observational studies and large-scale trials on the complex interactions between low-carbohydrate diets and long-term outcomes are needed.

## Introduction

A growing body of evidence has suggested that low-carbohydrate diets and their combination with high-protein diets are effective in weight loss. [1]–[3] In addition, they reportedly ameliorate the risk factors of cardiovascular disease (CVD) in the short term, [4]–[6] which would decrease incident CVD and mortality. However, recent cohort studies did not support this hypothesis [7]–[12] and their long-term health benefit and risk remain controversial. In fact, low-carbohydrate diets tend to result in reduced intake of fiber and fruits, and increased intake of protein from animal sources, cholesterol and saturated fat, all of which are risk factors for mortality and CVD. [13], [14].

In light of the worldwide obesity epidemic and the widespread popularity of low-carbohydrate diets, explorations of their long-term health outcome are of clinical importance for the control of weight. Moreover, they are crucial in the areas of public health, since a modest increase in the risk of morbidity and mortality [15] translates into a substantial social burden. These circumstances prompted us to investigate, with greater precision, the effects of low-carbohydrate diets on mortality and CVD incidence by scrutinizing pertinent original reports and combining their data in an attempt to obtain meaningful clues for the evaluation of benefit and harm associated with dietary modification.

## Methods

### Search

Searches of MEDLINE, EMBASE, ISI Web of Science, Cochrane Library, and ClinicalTrials.gov from their inception until September 12, 2012, were performed. Studies evaluating the risks of mortality or CVD incidence among subjects with low-carbohydrate intake, compared with those with high-carbohydrate intake, were identified using a combination of the following keywords: ‘low-carbohydrate diet’ or ‘carbohydrate-restricted diet’, and ‘mortality’ or ‘survival’, and ‘cardiovascular disease’. The reference lists of the pertinent articles were also inspected.

### Selection

We assessed all the identified studies on the effects of low-carbohydrate diets on mortality and CVD risk based on original data analyses to determine their eligibility for inclusion in a qualitative analysis. The inclusion criteria in the meta-analysis were as follows: a published full-text report, randomized controlled trials (RCTs) or observational studies of at least one year’s follow-up period, reporting relative risks, i.e. hazard ratios (HRs), risk ratios (RRs), or odds ratios with confidence intervals (CIs), adjusted for at least three of the following possible major confounders for CVD and death: age, gender, obesity, smoking status, diabetes, hypertension, hypercholesterolemia, prior history of CVD, and family history of CVD. Studies in which the low-carbohydrate/high-protein (LC/HP) score was utilized to evaluate the carbohydrate intake were also included.

### Validity and Quality Assessment

To ascertain the validity of the eligible studies, the quality of each report was appraised in reference to the CONSORT statement [16] and the STROBE statement [17] as appropriate. The quality of the studies that were included in the meta-analysis were further evaluated using Newcastle-Ottawa Scale [18] with a score of 5 or less (out of 8) indicating a high risk of bias.

### Data Abstraction

We reviewed each full-text report to determine its eligibility and extracted and tabulated all the relevant data independently. The extracted data included the characteristics of the subjects (including age, gender, and region), study design, published year, follow-up period, outcomes and the methods used for risk estimation. Any disagreement was resolved by a consensus among the investigators.

### Quantitative Data Synthesis

If more than one study was published for the same cohort with identical endpoints, the report containing the most comprehensive information on the population was included to avoid overlapping populations. The reports were summarized both qualitatively and quantitatively.

In the computation of the low-carbohydrate diet score, percentages of energy from protein and carbohydrate were divided into deciles. [7] For carbohydrate, the lowest decile received 10 points and the highest received 0 points, inversely. We pooled the relative risk in the highest score (lowest-carbohydrate intake) group with the lowest score (highest-carbohydrate intake) group as a referent. If an original article classified diets by the carbohydrate intake amount rather than the proportion to the total energy intake, the inverse relative risk for the lowest intake group was calculated with the highest intake group as a referent. If a relative risk was given per score in the original study, the relative risk in the highest score (lowest-carbohydrate intake) group was estimated by calculating the relative risk per score to the ninth power with the lowest score (highest-carbohydrate intake) group as a referent. Sensitive analysis was done using a composite LC/HP score. For protein, participants in the highest decile received 10 points, participants in the ninth decile received 9 points, and so forth. The protein and carbohydrate scores were then summed to create the composite LC/HP score (ranging from 2 to 20), which simultaneously assessed the position of each participant in terms of protein and carbohydrate intake. [9] Thus, a participant with a score of 2 was one with very high consumption of carbohydrates and very low consumption of proteins, whereas a participant with a score of 20 was one with very low consumption of carbohydrates and very high consumption of proteins. We pooled the relative risks similarly.

In the meta-analysis, each adjusted relative risk with low-carbohydrate intake was combined and the pooled RR with a 95% CI was calculated using the random-effects model with inverse-variance weighting. If a study separately reported relative risks for men and women, an overall estimate for the study was calculated from the two relative risks using the fixed-effects model with inverse-variance weighting and these single estimates were used in the subgroup analysis evaluating the individual contribution of the gender. [19] The results based on the LC/HP score were pooled separately. Heterogeneity among the studies was evaluated using I^2^ statistics. RevMan (version 5.1) was used for these calculations. All the procedures were in accordance with the guidelines for the meta-analysis of observational studies in epidemiology [20] and the PRISMA statement [21].

## Results

### Search Results

**Figure 1:**
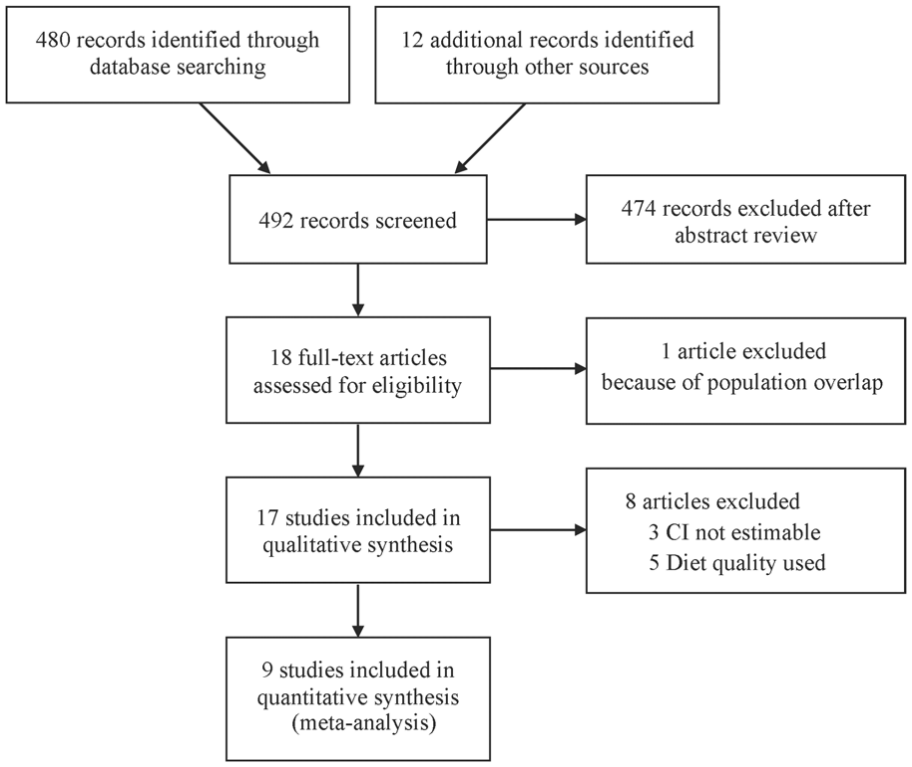
Flow diagram of study selection.

A total of 492 articles were identified during our search; of these, 18 were assessed with respect to their eligibility for inclusion in our review, which was aimed at determining the influence of low-carbohydrate diets on mortality and CVD incidence (**Fig. 1**). No RCTs were identified. One article [22] was excluded from the systematic review because of population overlapping. Out of these 18 articles, a total of 17 cohort studies [7]–[12], [14], [23]–[32] were included in the systematic review and meta-analysis.

**Table 1** shows the characteristics of each included study according to the published year. The 17 selected articles included in the systematic review were moderately heterogeneous in terms of population demographics, carbohydrate intake parameter, and the assessment of confounding factors. The population sample size in these studies ranged from 647 to 129,716. The majority of the articles were published from Sweden and the United States (US).

**Table 1:** Study characteristics.

Source	Country, region/cohort	Follow-up, yr	N (women, %)	Age, yr	Diabetes, %	Coronary heart disease, %	Outcome, n
Garcia-Palmieri, 1980* [23]	USA, Puerto Rico	6	8218 (0)	45–64	NR	0	Myocardial infarction or coronary heart disease death 286
McGee, 1984* [24]	USA, Japanese ancestry	10	7088 (0)	45–68	NR	0	Coronary heart disease 456
McCullough, 2000* [25]	USA, NHS	12	67272 (100)	45–64	0	0	All CVD 1427
McCullough, 2000* [26]	USA, HPFS	8	51529 (0)	40–75	0	0	All CVD 1092
McCullough, 2002* [14]	USA,	8–12					
	a. NHS		a. 67271 (100)	a. 30–55	0	0	a. All CVD 1365
	b. HPFS		b. 38615 (0)	b. 40–75	0	0	b. All CVD 1092
Fung, 2001* [27]	USA, NHS	12	69017 (100)	38–63	0	0	Coronary heart disease 821
Diehr, 2003* [28]	USA, US Cardiovascular Health Study	10	5888 (58)	73	11	25	Coronary heart disease 2179
Oh, 2005 [29]	USA, NHS	18	78779 (100)	30–55	0	0	All stroke 1020
							Ischemic stroke 515
							Hemorrhagic stroke 279
Halton, 2006 [30]	USA, NHS	20	82802 (100)	30–55	0	0	Coronary heart disease 1994
Beulens, 2007 [31]	Netherland, Prospect-EPIC	Mean 9	15714 (100)	49–70	0	0	All CVD 799
							Coronary heart disease 556
							Stroke 243
Lagiou, 2007 [11]	Sweden, Scandinavian Women’s Lifestyle and Health Cohort	Mean 12	42237 (100)	30–49	0	0	All-cause death 588
							CVD death 75
Massimino, 2007* [32]	Brazil, Japanese-Brazilians	8	647 (52)	Mean 63.5	20	NR	All-cause death 71
Trichopoulou, 2007 [12]	Greece, EPIC	Mean 4.9	22944 (59)	Adults	0	0	All-cause death 455
							CVD death 193
Fung, 2010 [7]	USA,						
	a. NHS	a. 26	a. 85168 (100)	a. 34–59	a. 0	a. 0	a. All-cause death 12555
							CVD death 2458
	b. HPFS	b. 20	b. 44548 (0)	b. 40–75	b. 0	b. 0	b. All-cause death 8678
							CVD death 2746
Sjögren, 2010 [8]	Sweden, Uppsala	Mean 10.1	924 (0)	Mean 71	0	0	All-cause death 215
							CVD death 88
Lagiou, 2012 [9]	Sweden, Uppsala Longitudinal Study of Adult Men cohort	Mean 15.7	43396 (100)	30–49	NR	0	All CVD 1268
							Ischemic heart disease 701
							Ischemic stroke 294
							Hemorrhagic stroke 70
							Subarachnoid hemorrhage 121
							Peripheral arterial disease 82
Nilsson, 2012 [10]	Sweden, Västerbotten Intervention Program	Median 10	77319 (51)	Median 49	3	NR	All-cause death 2383
							CVD death 681

NR: not reported, CVD: cardiovascular disease, LCHP: low-carbohydrate/high-protein,

* not included in meta-analysis, NHS: Nurses’ Health Study, HPFS: Health Professionals Follow-up Study, EPIC: European Prospective Investigation into Cancer and Nutrition.

The adjustment factors and the risk of bias among the studies are summarized in **Table 2** and **Table S1**, respectively. Major confounding factors such as total energy intake were not stated in two studies. [23], [32] Few inspected any updates of the carbohydrate intake over the follow-up period. Protein source was added to analysis in 3 studies. [7], [9], [30] The risk of bias among the researches involved in the meta-analysis was low.

**Table 2:** Methodological assessments of the included studies.

Source	Parameter	Outcome measures	Referent	Comparator	Adjustment factors
Garcia-Palmieri, 1980* [23]	Carbohydrate intake	Coefficient			Alcohol, systolic blood pressure, cholesterol, cigarettes smoked, and blood glucose
McGee, 1984* [24]	Carbohydrate intake	Coefficient			Energy intake, blood pressure, serum cholesterol, cigarettes smoked per day, body weight (in pounds), and physical activity index
McCullough, 2000* [25]	Healthy eating index-f	Relative risk	Quintile 5	Quintile 1	Age (5-y categories), smoking (never, past, 1–14 cigarettes/d, 15–24 cigarettes/d, or ≥25 cigarettes/d), time period, body mass index (quintiles), alcohol intake (7 categories), physical activity (6 categories of metabolic equivalents), history of hypertension or hypercholesterolemia at baseline, total energy intake (quintiles), postmenopausal status, postmenopausal hormone use, multivitamin and vitamin E supplement use
McCullough, 2000* [26]	Healthy eating index-f	Relative risk	Quintile 5	Quintile 1	Age (5-y categories), body mass index (quintiles), smoking (never, past, 1–14 cigarettes/d, 15–24 cigarettes/d, ≥25 cigarettes/d), alcohol intake (7 categories), physical activity (6 categories), total energy intake (quintiles), time period, multivitamin use, vitamin E use, and diagnosis of hypercholesterolemia and hypertension at baseline
McCullough, 2002* [14]	Recommended Food Score	Relative risk	Quintile 5	Quintile 1	Age (5-y categories), smoking (never, past, 1–14 cigarettes/d, 15–24 cigarettes/d, >25 cigarettes/d), time period, body mass index (quintiles), physical activity (6 categories of metabolic equivalents), total energy intake (quintiles), history of hypertension or hypercholesterolemia at baseline, vitamin E and multivitamin supplement, and for women, postmenopausal hormone use
Fung, 2001* [27]	Prudent pattern/Western pattern	Relative risk	Quintiles4,5/1	Quintiles 1/4,5	Age, period, smoking, body mass index, hormone replacement therapy, aspirin use, caloric intake, family history, history of hypertension, multivitamin and vitamin E use, and physical activity
Diehr, 2003* [28]	Diet quality	Years of lifein 10 yr,CVDincidence	Healthy diet	Unhealthy diet (high fat, low fiber, low carbohydrate, high protein, high calorie)	Demographics, health, behaviors, and baseline health variables
Oh, 2005 [29]	Carbohydrate intake	Relative risk	Quintile 5	Quintile 1	Age (5-year categories), body mass index (five categories), smoking (never, past, current 1–14, 15–24, ≥25 cigarettes/day), alcohol intake (four categories), parental history of myocardial infarction, history of hypertension, hypercholesterolemia, and diabetes, menopausal status and postmenopausal hormone use, aspirin use (five categories), multivitamin use, vitamin E supplement use, physical activity (hours/week, five categories), energy, cereal fiber (quintiles), saturated fat, monounsaturated fat, polyunsaturated fat, trans-fat, and omega-3 fatty acids (quintiles)
Halton, 2006 [30]	Low carbohydrate score	Relative risk	Decile 1	Decile 10	Age (in 5-year categories), body-mass index (<22.0, 22.0 to 22.9, 23.0 to 23.9, 24.0 to 24.9, 25.0 to 27.9, 28.0 to 29.9, 30.0 to 31.9, 32.0 to 33.9, 34.0 to 39.9, or ≥40.0), smoking status (never, past, or current [1 to 14, 15 to 24, or ≥25 cigarettes a day]), postmenopausal hormone use (never, current use, or past use), hours of physical activity per week (<1, 1 to 2, 2 to 4, 4 to 7, or >7), alcohol intake (0, <5 g per day, 5 to 14 g per day, or ≥15 g per day), number of times aspirin was used per week (<1, 1 to 2, 3 to 6,7 to 14, or ≥15), use of multivitamins (yes or no), use of vitamin E supplement (yes or no), history of hypertension (yes or no), history of hypercholesterolemia (yes or no), parental history of myocardial infarction (yes or no), and total calories
Beulens, 2007 [31]	Carbohydrate intake	HR	Quartile 4	Quartile 1	Age, hypertension, cholesterolemia, smoking (never/past/current smoking of 1 to 10, 11 to 20, and ≥20 cigarettes), body mass index, mean systolic blood pressure, total physical activity, menopausal status (pre or post), hormone replacement therapy use, oral contraceptives use, alcohol intake (≤10, 11 to 25, 26 to 50, ≥50 g/day energy-adjusted), total energy intake (in quintiles) and energy-adjusted intake of vitamin E, protein, dietary fiber, folate, saturated fat, and poly- and monounsaturated fat
Lagiou, 2007 [11]	Low carbohydrate score	HR	Per decreasing tenth of carbohydrate intake		Height (cm, continuously), body mass index (<25, 25–29.99 and 30 kg m2, categorically), smoking status (never smokers, former smokers of <10 cigarettes, former smokers of 10–14 cigarettes, former smokers of 15–19 cigarettes, former smokers of 20 or more cigarettes, current smokers of <10 cigarettes, current smokers of 10–14 cigarettes, current smokers of 15–19 cigarettes, current smokers of 20 or more cigarettes, categorically), physical activity [from 1 (low) to 5 (high), categorically], education (0–10, 11–13 and 14 or more years in school, categorically), energy intake (per 1000 kJ day), continuously), saturated lipid intake (per 10 g, continuously) and alcohol intake (<5, 5–25 or >25 g day, categorically).
	LCHP score	HR	Per increasing 2 points		
Massimino, 2007* [32]	Carbohydrate intake	HR	Tertile 3	Tertile 1	Gender (male/female), age (in years), generation (second versus first), physical activity (other versus heavy/very heavy), arterial pressure (systolic and diastolic, in mmHg), degree of glucose tolerance (“dummy”: normal glucose tolerance, altered fasting blood glucose, impaired glucose tolerance, and diabetes mellitus), presence of dyslipidemia (yes/no), and smoking (smoker/non-smoker)
Trichopoulou, 2007 [12]	Carbohydrate intake	HR	Per decreasing tenth of carbohydrate intake		Energy intake, gender (men, women; categorically), age (<45 years, 45–54 years, 55–64 years, ≥65 years; categorically), years of schooling (<6, 6–11, 12, ≥13; categorically), smoking (never, former and 1–10 cigs per day, 11–20 cigs per day, 21–30 cigs per day, 31–40 cigs per day, ≥41 cigs per day; ordered), body mass index (per quintile; ordered), physical activity (per quintile; ordered), and ethanol intake (<10 g per day, 10–30 g per day, ≥30 g per day; categorically).
	LCHP score	HR	Per increasing 2 points for CVD death		
			Lowest group (2–6 points) for all-cause death	Highest group (16–20 points)	
Fung, 2010 [7]	Low carbohydrate score	HR	Decile 1	Decile 10	Age, physical activity, body mass index, energy intake, alcohol intake, menopausal status and postmenopausal hormone use (women only), history of hypertension, smoking status, and multivitamin use.
Sjögren, 2010 [8]	LCHP score	HR	Lowest group (2–6 points)	Highest group (16–20 points)	Energy intake, smoking, social class, type 2 diabetes, the metabolic syndrome, lipid-lowering treatment, blood pressure–lowering treatment, waist circumference, diastolic blood pressure, insulin, C-reactive protein
Lagiou, 2012 [9]	Low carbohydrate score	HR	Per decreasing tenth of carbohydrate intake		Height (cm, continuously), body mass index (<25, 25–29.99, and ≥30, categorically), smoking status (never smokers, former smokers of <10 cigarettes, former smokers of 10–14 cigarettes, former smokers of 15–19 cigarettes, former smokers of ≥20 cigarettes, current smokers of <10 cigarettes, current smokers of 10–14 cigarettes, current smokers of 15–19 cigarettes, and current smokers of ≥20 cigarettes, categorically), physical activity (from 1 (low) to 5 (high), categorically), education (≤10, 11–13, and ≥14 years in school, categorically), diagnosis of hypertension (ever versus never), energy intake (per 1000 kJ/day, continuously), unsaturated lipid intake (per 10 g/day, continuously), saturated lipid intake (per 10 g/day, continuously), and alcohol intake (<5 g/day, 5–25 g/day, and >25 g/day, categorically)
	LCHP score	HR	Per increasing 2 points		
Nilsson, 2012 [10]	Carbohydrate intake	HR	Per decreasing tenth of carbohydrate intake		Age, body mass index, sedentary lifestyle, education, current smoking, intake of energy, alcohol, and saturated fat
	LCHP score	HR	Lowest group (2–8 points)	Highest group (14–20 points)	

CVD: cardiovascular disease, LCHP: low-carbohydrate/high-protein, HR: hazard ratio,

* not included in meta-analysis.

### Qualitative Summary

The all of the studies included in our analysis were methodologically good in quality. Regression coefficients of the multiple logistic model were provided in two articles [23], [24] and CI was not estimable in another report. [32] Five articles analyzed the risk by diet quality without quantifying carbohydrate intake. [14], [25]–[28] These 8 articles were not included in the subsequent meta-analysis. Most of the studies included in the systematic review were conducted in the US and European countries and their follow-up durations were long enough for the outcomes to occur. Although the majority of the enrolled subjects were middle-aged and free of such chronic comorbidities as diabetes and coronary heart disease, healthcare professionals dominated in the US cohorts, who may not truly represented the average population in the community.

All-cause mortality was assessed in 7 reports. Four cohort studies using the low-carbohydrate score [7], [10], [11], [32] and two using the LC-HP score [11], [12] showed a significant increase associated with low-carbohydrate diets (relative risk range 1.12–25.0). One diet quality study suggested 0.27 shorter years of life in 10 years, which was statistically significant. [28] Only two out of five studies demonstrated a significantly elevated risk of CVD mortality (relative risk range 2.17–3.52) evaluated by the LC-HP score. [11], [12] One article showed a significantly elevated risk of CVD incidence estimated by the low-carbohydrate score and the LC-HP score (relative risk range 1.42–1.55), [9] whereas three diet quality researches suggested a significantly increased risk of incident CVD (relative risk range 1.30–1.56). [14], [26], [27] Neither of the studies that calculated regression coefficients showed a significant correlation between low-carbohydrate diets and CVD. [23], [24] Some studies suggested that low-carbohydrate diets might increase the risk of mortality and CVD in animal-based dietary patterns whereas they might decrease the risk in plant-based diets. [7], [9], [30].

The estimates in all the other analyses using either score were non-significant and none of these studies revealed that low-carbohydrate diets were associated with a significantly decreased risk of these outcomes.

### Quantitative Summary (Meta-analysis)

**Figure 2:**
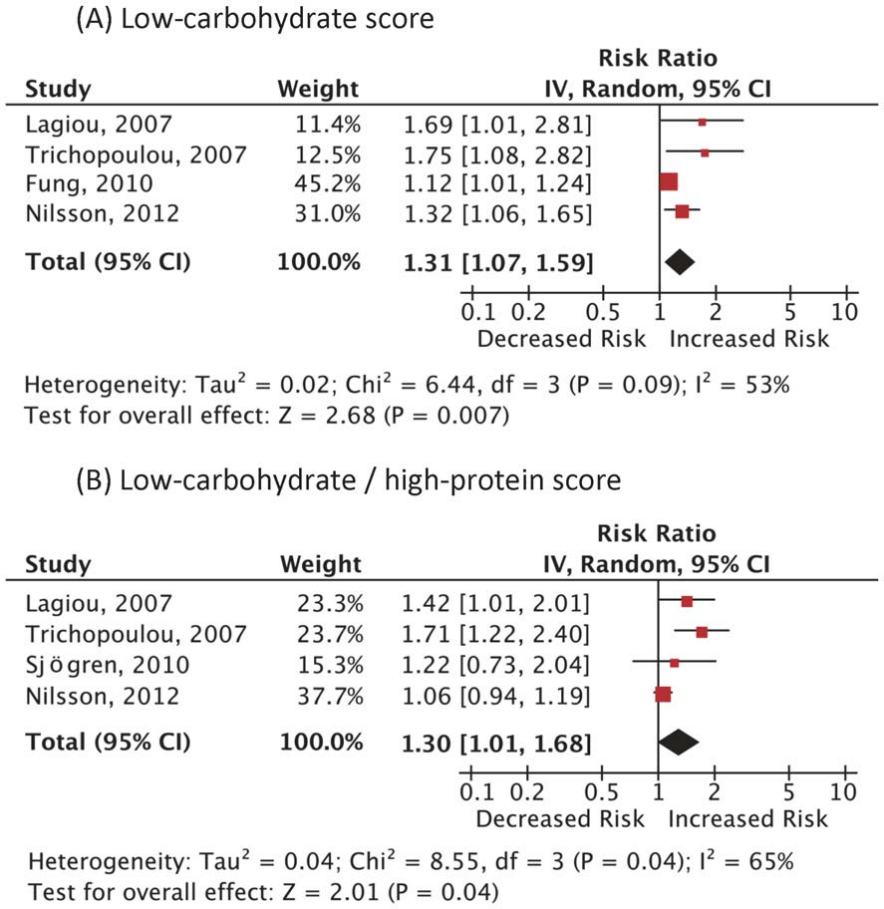
Adjusted risk ratios for all-cause mortality associated with low-carbohydrate diets. Analysis was done based on (A) the low-carbohydrate score and (B) the low-carbohydrate/high-protein score. Boxes, estimated risk ratios (RRs); bars, 95% confidence intervals (CIs). Diamonds, random-effects model RRs; width of diamonds; pooled CIs. The size of each box is proportional to the weight of each study in the meta-analysis. IV, inverse-variance.

A total of 9 articles that provided sufficient information using the low-carbohydrate score and/or the LC-HP score were included in the meta-analysis (**Fig. 1**). All the ascertainment of diagnosis was based on the valid registries but only a few specified the diagnostic criteria for CVD. [12], [29], [30] The follow-up rate was more than about 90% in each study. Carbohydrate intake was assessed by the residual method in 5 studies [8]–[12] and by the density method in 4 studies. [7], [29]–[31] Of the 272,216 people in 4 cohort studies using the low-carbohydrate score, 15,981 (5.9%) cases of death from all-cause were reported. **Fig. 2** illustrates the significantly increased risk of all-cause mortality among those adherent to low-carbohydrate diets: the pooled RR (95% CI) 1.31 (1.07–1.59); p = 0.007; I^2^ = 53% (p = 0.09). Analysis using the LC/HP score yielded a similar significant increase in the risk of all-cause mortality: RR 1.30 (1.01–1.68); p = 0.04; I^2^ = 65% (p = 0.04). A dose-response was observed in 2 analyses. [7], [12] Since heterogeneity among reports in the all-cause mortality using the low-carbohydrate score was statistically significant, we conducted a subgroup analysis according to the possible predictors. The pooled RRs of the studies conducted in Europe [10]–[12] and the United States [7] (RR 1.42 [1.18–1.72] vs 1.12 [1.01–1.24]) were both significantly elevated; and the diet assessment method (residual method [10]–[12] or density method [7]) coincided with these regions; the studies with follow-up length shorter than 10 years [10], [12] were associated with a statistically high RR while those with follow-up length longer than 10 years [7], [11] were not (RR 1.40 [1.12–1.74] vs 1.27 [0.88–1.84]); The pooled RR for men [7], [10] was statistically elevated while that for women [7], [9], [10] was not (RR 1.19 [1.08–1.31] vs 1.34 [0.96–1.87]). We were unable to perform a subgroup analysis according to the body-mass index because the mean values were not stated or estimable in the majority of the reports.

**Figure 3:**
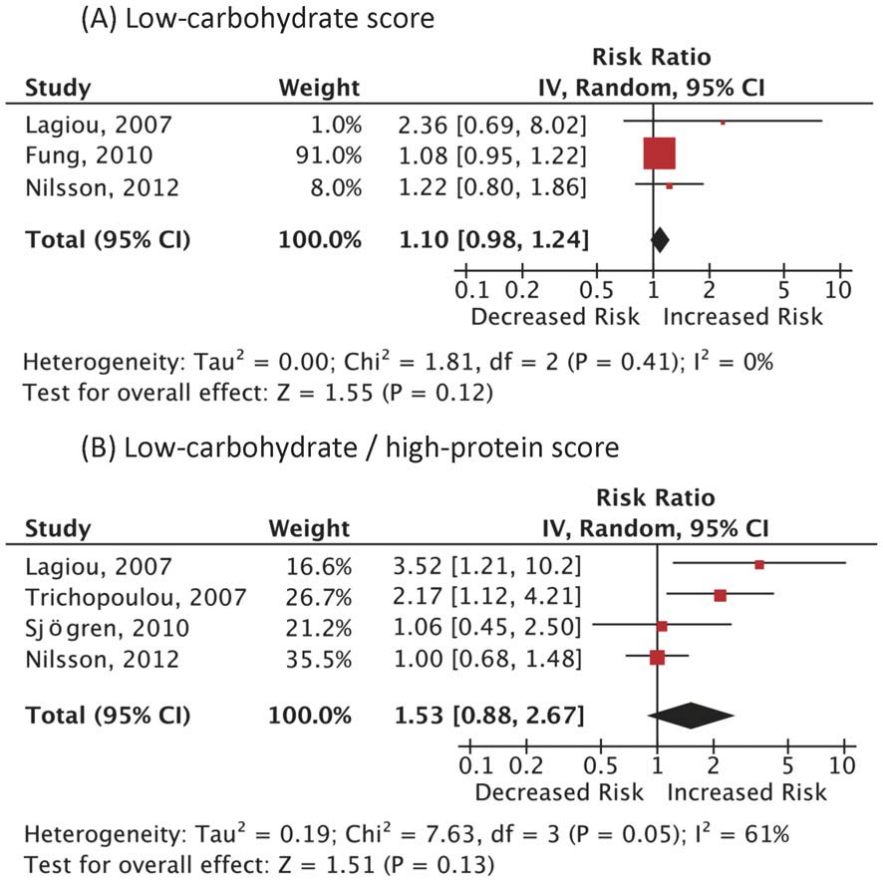
Adjusted risk ratios for CVD mortality associated with low-carbohydrate diets. Analysis was done based on (A) the low-carbohydrate score and (B) the low-carbohydrate/high-protein score. Boxes, estimated risk ratios (RRs); bars, 95% confidence intervals (CIs). Diamonds, random-effects model RRs; width of diamonds; pooled CIs. The size of each box is proportional to the weight of each study in the meta-analysis. IV, inverse-variance.

**Figure 4:**
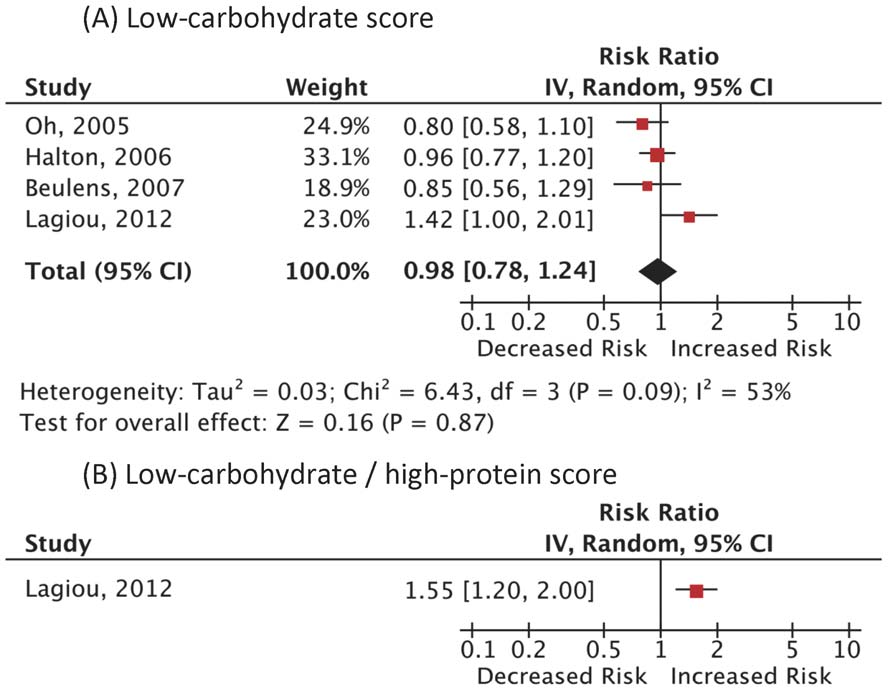
Adjusted risk ratios for CVD incidence associated with low-carbohydrate diets. Analysis was done based on (A) the low-carbohydrate score and (B) the low-carbohydrate/high-protein score. Boxes, estimated risk ratios (RRs); bars, 95% confidence intervals (CIs). Diamonds, random-effects model RRs; width of diamonds; pooled CIs. The size of each box is proportional to the weight of each study in the meta-analysis. IV, inverse-variance.

A total of 3,214 (1.3%) cases of CVD death among 249,272 subjects in 3 cohort studies and 5,081 (2.3%) incident CVD cases among 220,691 women in different 4 cohort studies were reported. As summarized in **Fig. 3** and **Fig. 4**, the RRs of CVD mortality and incidence were not statistically significant: RR 1.10 (0.98–1.24); p = 0.12; I^2^ = 0% (p = 0.41), RR 0.98 (0.78–1.24); p = 0.87; I^2^ = 53% (p = 0.09), respectively. The RR in CVD mortality using the LC/HP score was not statistically significant, either: RR 1.53 (0.88–2.67); p = 0.13; I^2^ = 61% (p = 0.05). There was only one study on CVD incidence using the LC/HP score, which showed a significantly elevated risk. [9] There was a positive dose-response in 2 analyses. [7], [9].

## Discussion

Our systematic review and meta-analyses of worldwide reports suggested that low-carbohydrate diets were associated with a significantly higher risk of all-cause mortality in the long run. They also suggested that low-carbohydrate diets might not be protective or harmful in terms of CVD mortality and incidence. These findings support the hypothesis that the short-term benefits of low-carbohydrate diets for weight loss are potentially irrelevant. [13] In light of the fact that the number of people with obesity is exponentially increasing worldwide and obesity is one of the leading risk factors of mortality, [15] our findings have substantial clinical and public implications on a global scale and point to the need for the further investigation of the long-term health effects of low-carbohydrate diets and other nutritional factors.

The strength of our present study is that the analysis was mainly based on long-term large population-based data originating from multiple nations and was performed with a high level of precision and this is the first meta-analysis, to our best knowledge, on the health effects of low-carbohydrate diets. The included data were good in quality and apparently had power enough to detect the differences in the risk of these outcomes. The outcome ascertainment tools were valid, and each result was adjusted for multiple confounders and the significantly increased pooled RRs for all-cause mortality were robust in that the RRs based on both of the methods were almost identical and statistically significant. Heterogeneity of the results of the component studies was modest: low heterogeneity suggests that the each result was consistent and most variation was attributable to chance alone, and the large I^2^ values in some analyses indicated that the range of the plausible risk estimates was wide, generally because of the diversity of study design, population backgrounds and ethnicities. The subgroup analysis suggested that the possible major source of heterogeneity was the region or the nutrition assessment method in addition to the publication bias. The main dietary source of protein and the obesity prevalence differ across countries [33]. The length of follow-up and the gender were possibly other sources of heterogeneity but these hypotheses cannot be statistically tested in light of the scarcity of data.

Evidence has been accumulating to suggest that low-carbohydrate diets and their combination with high-protein diets are effective in weight loss [1]–[3] and may have favorable short-term effects on the risk markers of CVD. [4]–[6] Low-carbohydrate diets may be nutritionally safe and valid insofar as the carbohydrates are simple and refined, and the main source of the protein is plants. Despite these facts, our study did not find a cardiovascular benefit and supports their potential long-term health harm when such nutritional quality is not considered. Low-carbohydrate diets tend to result in reduced intake of fiber and fruits, and increased intake of protein from animal sources, cholesterol and saturated fat, [27], [30], [34] all of which are risk factors for mortality and CVD. [13], [14] It is postulated that differences in dietary bioactive components such as specific free fatty acids, protein, fiber, minerals, vitamins and phytochemicals are involved. [7] Subgroup analyses suggested that low-carbohydrate diets might increase the risk of mortality and CVD in animal-based dietary patterns whereas they might decrease the risk in plant-based diets. [7], [9], [30] In our analysis, the increment in the all-cause mortality might have been partly attributable to the increased risks for CVD mortality and morbidity although they were not significant. It is possible that the beneficial effect of plant protein may have been offset by the adverse effect of animal protein in our calculations. Low-carbohydrate diets may be linked to an array of other chronic health problems. A positive cancer risk has been reportedly related to the intake of animal protein, [7] and red and processed meat consumption, [35] although the risk of cancer was found to be non-significant in our analysis. [11], [12] Little is known about the consequences of low-carbohydrate diets with respect to kidney disease, osteoporosis, and mental condition. The biology that underlies the positive correlation between low-carbohydrate diets and all-cause death is not fully explained. Further studies to clarify the mechanism are eagerly awaited.

Given the facts that low-carbohydrate diets are likely unsafe and that calorie restriction has been demonstrated to be effective in weight loss regardless of nutritional composition, [36] it would be prudent not to recommend low-carbohydrate diets for the time being. Further detailed studies to evaluate the effect of protein source are urgently needed.

### Limitations

Although the quality of the included studies might not be an issue, our analysis should be interpreted in the context of the following limitations. The observational studies were scarce and moderately heterogeneous, and thus a publication bias and a residual confounding bias may have existed although we cannot assess these hypotheses. In the analysis of CVD mortality risk, there may not have been enough statistical power and the representativeness of the cohort may be poor since the data of healthcare professionals [7] dominated (**Fig. 3A**). Next, the relation may not necessarily be causal, particularly in the observational studies [37] because of possible confounding factors and biases that may not have been fully adjusted for, which may have rendered the results less valid. In our analysis, the adjustment in each component study was adequate and fair. Confounding by treatment indication [38] might bias the effect of diets. However, most of the target populations were free of chronic disease at baseline and it is less likely that the dietary habits had been modulated according to their previous health status. A dose-response of relative risk was confirmed in few studies, which might make the results less plausible. Dietary patterns may vary over the course of follow-up but updating dietary information was not done in many studies and thus the magnitude of risk may have been diluted as suggested by our subgroup analysis of the flow-up periods and the supplementary analysis by Lagiou, et al. [9] Furthermore, it is difficult to distinguish the effects of individual nutritional component. For all these limitations, however, observational studies provide good available evidence regarding potential benefit and harm, and the overall pooled estimates were robust, the temporal sequence of the events was appropriate, and the results among the included studies seemed consistent. Moreover, evidence has been accumulating to support these potential adverse outcomes. [39] With regards to external validity, it is also important to realize that the participants of the studies may not represent general populations most likely because the majority of the studies were done in Western countries and healthcare professionals dominated. It remains unclear if these diets exert a similar influence on the clinical outcome in diabetic patients.

Even with these limitations, none of the included studies showed a significantly reduced risk and our analysis does not favor long-term benefits of low-carbohydrate diets, which should provide physicians with an incentive to pay attention to the considerable potential adverse effects on health if such diets are implemented without considering the nature of the carbohydrates and the source of protein. [9].

### Conclusions

Our meta-analysis supported long-term harm and no cardiovascular protection with low-carbohydrate diets. However, the observational studies were limited and moderately heterogeneous. Our findings underscore the imminent need for large-scale trials on the complex interactions between low-carbohydrate diets and long-term outcomes.

## Supporting Information

Table S1 **Newcastle-Ottawa quality assessments of the included studies.**
(DOCX)
