# Correction: Low-Carbohydrate Diets and All-Cause Mortality: A Systematic Review and Meta-Analysis of Observational Studies

**DOI:** 10.1371/journal.pone.0212203

**Published:** 2019-02-07

**Authors:** Hiroshi Noto, Atsushi Goto, Tetsuro Tsujimoto, Mitsuhiko Noda

The authors wish to correct the number of all-cause deaths in the Abstract and Results section of [1]. The correct number of all-cause deaths is 24,659 (9.1%), rather than 15,981 (5.9%). As a result, the following sentences need to be corrected:

In the Abstract, the second sentence of the “Results” subsection should read: “Of the 272,216 people in 4 cohort studies using the low-carbohydrate score, 24,659 (9.1%) cases of death from all-cause were reported.”

In the Results, the fifth sentence of the “Quantitative Summary (Meta-analysis)” subsection should read: “Of the 272,216 people in 4 cohort studies using the low-carbohydrate score, 24,659 (9.1%) cases of death from all-cause were reported.”

The authors confirm that this error does not affect the conclusions.
