# Supplementary material for: Low-Carbohydrate Diets and All-Cause Mortality: A Systematic Review and Meta-Analysis of Observational Studies
Source: PLoS One. 2013 Jan 25;8(1):e55030. doi: 10.1371/journal.pone.0055030 (PMC3555979; doi:10.1371/journal.pone.0055030)
Supplement: Table S1 — Newcastle-Ottawa quality assessments of the included studies. (DOCX) [file pone.0055030.s001.docx]

**Table S1. Newcastle-Ottawa quality assessments of the included studies.**

| **Study** | **Selection** |  |  |  | **Comparability** | **Outcome** |  |  | **Total quality score** |
| --- | --- | --- | --- | --- | --- | --- | --- | --- | --- |
|  | **(1) Representativeness of the exposed cohort** | **(2) Selection of the non-exposed cohort** | **(3) Ascertainment of exposure** | **(4) Demonstration that outcome of interest was not present at start of study** | **(1) Comparability of cohorts on the basis of the design or analysis^a^** | **(1) Assessment of outcome** | **(2) Was follow-up long enough for outcomes to occur?^b^** | **(3) Adequacy of follow up of cohorts^c^** |  |
| Oh, 2005 [29] |  | ★ | ★ | ★ | ★★ | ★ | ★ | ★ | 8 |
| Halton, 2006 [30] |  | ★ | ★ | ★ | ★★ | ★ | ★ | ★ | 8 |
| Beulens, 2007 [31] | ★ | ★ | ★ | ★ | ★★ | ★ |  | ★ | 8 |
| Lagiou, 2007 [11] | ★ | ★ | ★ | ★ | ★★ | ★ | ★ | ★ | 9 |
| Trichopoulou, 2007 [12] | ★ | ★ | ★ | ★ | ★★ | ★ |  | ★ | 8 |
| Fung, [7] |  | ★ | ★ | ★ | ★★ | ★ | ★ | ★ | 8 |
| Sjögren, 2010 [8] | ★ | ★ | ★ | ★ | ★★ | ★ | ★ | ★ | 9 |
| Lagiou, 2012 [9] | ★ | ★ | ★ | ★ | ★★ | ★ | ★ | ★ | 9 |
| Nilsson, 2012 [10] | ★ | ★ | ★ | ★ | ★★ | ★ | ★ |  | 8 |

**a: A maximum of 2 stars can be awarded for this item. A study controlling for age receives one star, and a study controlling for other major risk factors for CHD and death receives an additional star.**

**b: A study with a follow-up period ≥10 years receives one star.**

**c: A study with a follow-up rate >90% receives one star.**
